# Perioperative care of a patient with stroke

**DOI:** 10.1186/1755-7682-3-33

**Published:** 2010-11-20

**Authors:** Tonny V Veenith, Asmat H Din, Danielle MJ Eaton, Rowan M Burnstein

**Affiliations:** 1 Division of Anaesthesia, University of Cambridge, Cambridge, CB2 2QQ, UK

## Abstract

Strokes and TIAs, with their high cumulative mortality and morbidity rates, are occurring with increasing frequency in western population [1,4]. As such, it is vital for clinicians to provide optimal medical management in the perioperative period for those patients with this common neurological problem. This review aims to highlight the importance of the perioperative period and the stages of pre-optimization that can be taken by the multi-disciplinary team to aid this [17-19]. The evidence suggests that there are significant physiological advantages to early invasive monitoring and high dependency care in these complex patients. These cohort of patients are at increased risk of development of respiratory, gastrointestinal, nutritional and electrolyte disturbances so a constant vigil should be exercised in early recognition and treatment.

## Introduction

Peri- and post-operative care of the patient with neurologic illness poses a clinical and logistical challenge for the team participating in the care of the patient. The management of patients with cerebrovascular disease undergoing surgery requires an understanding of the pathophysiological mechanisms involved. This enables appropriate selection of patients who are suitable for surgery, with pre-optimization and vigilance to avoid predictable complications, as well as continued evaluation to detect any peri-operative deterioration.

### A) Stroke

A Transient Ischaemic Attack (TIA) occurs when there is a change to the blood supply of an area of the brain resulting in a change in neurologic function lasting for less than 24 hours. If the neurological dysfunction persists for up to 72 hours, but resolves, this is termed a Reversible Ischaemic Neurological Defect (RIND). When the neurological dysfunction persists for more than 72 hours, it is categorised as a stroke.

#### i) Ischaemic and haemorrhagic stroke (CVA)

**Figure 1:**
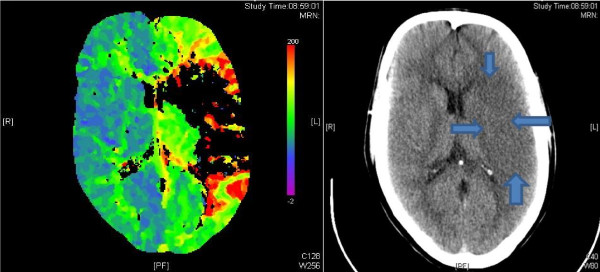
**Showing a perfusion scan and a CT scan of a patient with a Left middle cerebral artery infarct **[12]**[modified from reference **[13]].

Stroke is the third leading cause of death and affects 5.1% of the general population in the United States. In Europe the incidence ranges from 101 to 239 per 100000 population [1,2]. Strokes commonly can be subdivided into haemorrhagic or ischaemic; such as the middle cerebral artery occlusion and infarct illustrated in Figure 1. The incidence of stroke increases with age; with over 75% occurring after the 65 years of age. Ischaemic stroke contribute to 87% of all strokes. The rest (13%) are accounted for by non-traumatic haemorrhagic strokes [3]. Hemorrhagic strokes can be further divided into intracerebral haemorrhage and subarachnoid haemorrhage (SAH). Unlike ischaemic strokes, haemorrhagic strokes are not associated with transient ischaemic attacks. The cumulative mortality for the ischaemic stroke at five years after first-ever stroke is 60.1% [4]. The overall mortality for the haemorrhagic strokes is high when compared to the ischaemic stroke and the risk of mortality is up to 52% in first two days [5,6].

When evaluating a patient preoperatively with ischaemic or haemorrhagic stroke the following should be considered -

[1] Cause of the stroke (Table 1)

**Table 1:** Underlying mechanisms of haemorrhagic and ischaemic stroke 3

Ischaemic stroke	Haemorrhagic stroke
Thrombus - from a ulcerated plaque in the carotid artery	Hypertension is the commonest - causing weakness of the vessel wall

Cardioembolism from Atrial fibrillation (AF)	Amyloid angiopathy

Large vessel atherosclerosis causing a stroke by occlusion	Use of sympathomimetic drugs such as cocaine causing transient hypertension

Small deep perforating vessel arthrosclerosis causing lacunar stroke	Congenital Arterio-venous malformations, aneurysms

A venous clot, causing paradoxical embolism through a patent foramen ovale (PFO)	Uncommon such as tumours, vasculitis, bleeding diathesis and use of anticoagulants

Vasospasm of structurally normal vessels (e.g. sympathomimetic drug use)	

Hypercoagulable state, operating with any of these mechanisms or independently	

[2] Patient risk factors (Table 2)

**Table 2:** Risk factors for the development of stroke 3578910

Ischaemic stroke	Haemorrhagic stroke
***Common traditional factors*** Hypertension, previous transient ischaemic attacks, Smoking, Diabetes Mellitus, AF, Cardiovascular diseases (MI, angina, Peripheral vascular diseases) and heart failure	***Common traditional factors*** Male sex, age, hypertension, diabetes mellitus and alcohol intake.

***Haemostatic factors ***- factor VIIIc and von Willebrand factor	***Haemostatic factors ***- *Use of the anticoagulants and antiplatlet agents*

***Non traditional factors ***- Waist hip ratio, Left ventricular hypertrophy, Apolipoprotein(a) levels and White cell counts suggestive of inflammation	***Non traditional factors - ****Previous TIA's*

[3] Prognosis of the patient - is the surgery appropriate? (Tables 3 and 4)

**Table 3:** Predictors of death for the haemorrhagic (predicting death and disability at 30 days) and ischaemic strokes (death and disability at 100 days) 611

	Haemorrhagic stroke	Ischaemic stroke
Size and location	The volume of ICH and location	Right and left arm paresis at admission, lenticulostriate infarction

Premorbid status		Age, sex, prior stroke and Diabetes

Severity at presentation	The Glasgow Coma Scale (GCS) on admission	National institutes of health Stroke Scale at admission and Rankin Scale 48-72 hours later

Complications	Hydrocephalus	Fever, neurological complications.

**Table 4:** Favourable predictors of ischaemic and haemorrhagic stroke 4

Haemorrhagic stroke	Ischaemic stroke
Cortical location	Location and good function at presentation

Mild neurological dysfunction	Mild neurological dysfunction

Low fibrinogen levels	Absence of AF as this predicts more Cardiovascular deaths

[4] Patient risk factors for development of death following a TIA (Table 5)

**Table 5:** Risk factors increasing mortality following TIA

*Risk factors for development of death following a TIA*
Previous strokes

Stroke in heavy smokers - > 40 per day (level comes to normal after 5 years of stopping)

Atrial Fibrillation -increasing risk about 5-fold

Diabetes, hypertension, MI and high cholesterol

Race - African American race has higher incidence

Conjugate equine oestrogen increased risk of ischemic stroke and TIAs by 55%

#### ii) Stroke: Transient ischaemic attacks

Transient ischaemic attacks are much more common than strokes and occur in up to one in fifteen patients over 65 years [13]. The 90 day risk for a stroke after a TIA is 3% to 17.3% and is at its highest within the first 30 days after a TIA. Approximately half of patients who experience a TIA fail to report it to their healthcare providers [14-16]. The risk of dying after TIA is highest in the first 12 months.

### Prevention of ischaemic strokes and the transient ischaemic attacks

The management strategies for the prevention of ischaemic strokes and infarcts are summarised in table 6[17-19]. Prevention of the haemorrhagic stroke is achieved by the reduction of contributing risk factors. Addressing all secondary prevention strategies decreases the risk of further stroke, and as such, should be taken into account during pre-operative assessment.

**Table 6:** Reduction strategies for the prevention of TIA and ischaemic strokes

Strategy		Evidence
**Risk factor management**	Hypertension	Class I, Level of Evidence A, Benefit has been associated with an average reduction of &10/5 mm Hg (Normal defined by JNC 7 criteria as 120/80 mmHg). Drugs should be optimised to the target patient (consider the cardiac history, DM etc)
	
	Diabetes mellitus	Class I, Level of Evidence A, Rigorous control of hypertension and intensive lipid lowering treatment, any drugs appropriate and most often > 1 drug needed.
	
	Hypercholesterolemia	Class I, Level of Evidence A, Lifestyle modification, dietary guidelines and statins
	
	Cigarette Smoking	Class I, Level of Evidence C, Strongly advise every patient with stroke or TIA to quit
	
	Alcohol Intake	Class I, Level of Evidence A. Heavy drinkers should eliminate or reduce their consumption of alcohol.(it is actually a J-shaped association between alcohol and ischemic stroke, with a protective effect in light or moderate drinkers and an elevated stroke risk with heavy alcohol consumption)
	
	Obesity	Class IIb, Level of Evidence C. BMI of between 18.5 and 24.9 kg/m^2 ^and a waist circumference of < 35 in for women and < 40 in for men
	
	Physical inactivity	Class IIb, Level of Evidence C. At least 30 minutes of moderate-intensity physical exercise

**Interventional Approaches**	Carotid endartrectomy(CEA)	Class I, Level of Evidence A. TIA or ischemic stroke within the last6 months and severe (70% to 99%) carotid artery stenosis, CEA by a surgeon with a peri-operative morbidity and mortality of < 6%. Consider CEA for certain high risk candidates with moderate stenosis (50-99%). If the TIA preferably within 2 weeks of after TIA. Carotid angioplasty and stenting may be suitable for some patients with symptomatic high-grade stenosis and factors that make CEA unfavourable.
	
	Extracranial Vertebrobasilar Disease/Intracranial Atherosclerosis	Class IIb, Level of Evidence C Endovascular treatment - when patients are having symptoms despite medical therapies

**Medical Treatments**	Cardiogenic Embolism and PAF	Class I, Level of Evidence A, paroxysmal (intermittent) AF, anticoagulation with adjusted-Dose Warfarin (target INR, 2.5; range, 2.0 to 3.0). Aspirin to be given in those patients with a clear contra-indication to Warfarin.
	
	Cardiogenic Embolism - LV thrombus	Oral anticoagulation is reasonable, aiming for an INR of 2.0 to 3.0 for at least 3 months and up to 1 year (Class IIa, Level of Evidence B)
	
	Noncardioembolic ischemic stroke or TIA	(Class IIa, Level of Evidence A) Aspirin (50 to 325 mg/d), the combination of aspirin and extended-release dipyridamole, and clopidogrel are all acceptable options for initial therapy. Aspirin to clopidogrel increases the risk of haemorrhage and is not routinely recommended for ischemic stroke or TIA patients (Class III, Level of Evidence A).
	
	Patent Foramen Ovale	PFO closure may be considered for patients with recurrent cryptogenic stroke despite optimal medical therapy (Class IIb, Level of Evidence C)

### Initial assessment and treatment of ischaemic strokes [18]

Patients who have an acute TIA or stroke with a good recovery should be commenced on appropriate antiplatlet therapy and risk factors such as hypertension should be dealt with promptly prior to referral to a specialist neurovascular clinic. If there is evidence of more than one TIA in 24 hours, ideally the patient should be admitted to hospital and investigated as an inpatient.

Initial neurological assessment should focus on the area of the brain affected and imaging of the brain (typically CT) should be arranged as soon as possible (< 24 hours). Imaging should be performed as an emergency if there are any suggestions of high risk features such as anticoagulant treatment, bleeding tendency and depressed level of consciousness including unexplained progressive or fluctuating symptoms. Other worrying features include papilloedema, neck stiffness or fever and severe headache at onset. Indications for thrombolysis or early anticoagulation, which is discussed below, must be considered. Furthermore, in addition to routine the observations, which should include conscious level, blood pressure, pulse, heart rhythm, temperature, oxygen saturation and hydration; the patients should be screened for evidence of aspiration/swallowing difficulties. Baseline investigations should include a full blood count, clotting screen, blood sugar, urea and electrolytes and an electrocardiogram.

#### Indications for thrombolysis

Thrombolysis (e.g. alteplase) should be performed after consultation with an experienced physician and within three hours of the onset of imaging confirmed ischaemic stroke (Class I, Level of Evidence); intra-arterial thrombolysis should be reserved for centres with interventional neuroradiology services.

### Acute treatment of intracerebral bleeds

Surgical intervention should be considered in cases of supratentorial haemorrhage with mass effect or posterior fossa/cerebellar haematoma. Neurosurgical opinion should be sought for cases of secondary hydrocephalus following the intracerebral bleed.

### Acute treatment of Subarachnoid Haemorrhage (SAH)

Whilst trauma remains the commonest cause of SAH, ruptured intracranial aneurysms account for 80% of non-traumatic cases. However, it remains standard, that regardless of cause, all cases should be referred for neurosurgical consideration unless the patient is for palliative treatment only. SAH is treated with either endovascular coiling or an open clipping (depending on the centre experience and availability of interventional neuroradiologists/neurosurgeons). Acute complications such as hydrocephalus may require an external ventricular drain to stabilise the patient and assess potential for neurological recovery. A detailed discussion is outside the scope of this review [20].

### Perioperative implications for patients with a history of stroke [21]

Patients with a history of stroke frequently have coexisting vascular disease. Therefore, the overall management aim is protection against further strokes as well as prevention of further damage to other organs.

These patients mostly have multiple vessel involvement including the coronary arteries. They are also likely to suffer from other co morbidities such as hypertension and/or diabetes mellitus. Chronic hypertension shifts the cerebral autoregulation curve to the right, rendering the patients vulnerable to the haemodynamic compromise and the stress associated with the surgery [12]. Relative hypotension may increase the risk of end organ ischaemia. To avoid these complications invasive monitoring may be necessary in the perioperative period with consideration for high dependency care postoperatively.

### Complications following the illness

Care is required to reduce the incidence of complications potentiated by the presence of focal neurological deficits, as well as those related to coexisting illnesses.

#### Respiratory complications following a CVA [22]

Alterations of respiratory control, mechanics, and pattern are common following a CVA and lead to gas exchange abnormalities. Central or obstructive sleep apnoea may occur. Immobility can cause further complications such as hypostatic or aspiration pneumonia and venous thromboembolism. Many of these patients would need admission to a critical care areas following surgery for these reasons.

#### GI complications [23]

These patients may be at increased risk of aspiration due to impaired swallowing, ineffective cough and reduced gastric emptying.

#### Nutrition [24]

Poor nutritional intake following the stroke may lead to malnutrition and may be associated with poor survival and functional outcomes after the surgery.

#### Dyselectrolemia

Strokes are notoriously associated with electrolytic disturbances for a variety of reasons. These include poor nutritional intake [24], dyselectrolemia directly related to the stroke [25] (acutely and associated cardiac arrhythmias) and diuretics [26] used for the treatment of hypertension.

#### Drug therapy and perioperative period

The team should be mindful of the hypercoagulable state created by the withdrawal of the drugs (such as statins and antithrombotic antiplatlet aggregation drugs) prior to surgery. This is often necessary to minimise surgical complications related to bleeding, but may also occur as a result of fasting. The risks/benefits of withdrawing such drugs need to be considered on a patient by patient basis.

## Conclusion

Stroke is the third leading cause of death in Europe [1] and has a cumulative mortality at 5 years of 60.1 [4]. A TIA carries a 3-17.3% risk of subsequent stroke within 90 days [14]. As well as mortality strokes confer a significant morbidity.

A careful approach must be taken to the evaluation of a patient who has had a previous stroke in determining whether they are fit for an unrelated surgical procedure. A multidisciplinary approach is required to first ascertain whether surgery is the most appropriate treatment option. This must take into account the estimated morbidity and mortality, the post-operative facilities available for rehabilitation, and of course the patient's wishes.

Pre-optimization is desirable in patients undergoing elective procedures. Following a stroke or TIA all relevant secondary prevention strategies should be addressed as per the latest American Heart Association (AHA)/American Stroke Association (ASA) guidelines of October 2010 [19]. This should include risk factor management, medical therapies, and surgical interventions as appropriate.

Patients with a history of stroke tend to have co-existing vascular disease and are more likely to suffer from co-morbidities like diabetes mellitus, hypertension, and ischaemic heart disease. The peri-operative team should aim to protect the patient from risk of further stroke, as well as to limit any further organ damage from hypoperfusion in these high-risk individuals. As such invasive monitoring is desirable, and consideration should be made to possible high-dependency care post-operatively.

Complications secondary to stroke must be appreciated and addressed in the post-operative period. These include the risk of venous thromboembolism related to decreased mobility associated with stroke, which will be potentiated by most surgical procedures. Stroke patients also have an increased risk of aspiration pneumonia [23,24]. Poor nutritional intake following the stroke may lead to malnutrition and may be associated with poor survival and functional outcomes after the surgery. Electrolyte abnormalities are commonly associated with strokes and may lead to dysrhythmias both peri- and post-operatively [24,25]. If anti-platelet drugs are withdrawn for surgical considerations the team should be aware of the increased risk of thrombosis.

The patient with a history of stroke undergoing surgery is high-risk, and requires careful pre-operative evaluation, pre-optimaztion, and close monitoring for predictable post-operative complications.

## Competing interests

The authors declare that they have no competing interests.

## Authors' contributions

TV, AD, DE and RB were involved in drafting the manuscript. All authors read and approved the final manuscript.
